# A fluorometric assay for trehalose in the picomole range

**DOI:** 10.1186/1746-4811-9-21

**Published:** 2013-06-20

**Authors:** Petronia Carillo, Regina Feil, Yves Gibon, Namiko Satoh-Nagasawa, David Jackson, Oliver E Bläsing, Mark Stitt, John Edward Lunn

**Affiliations:** 1 Dipartimento di Scienze e Tecnologie Ambientali Biologiche e Farmaceutiche, Seconda Università degli Studi di Napoli, Via Vivaldi 43, I-81100, Caserta, Italy; 2 Max Planck Institute of Molecular Plant Physiology, Am Mühlenberg 1, Potsdam-Golm, 14476, Germany; 3 INRA Bordeaux, University of Bordeaux, UMR1332 Fruit Biology and Pathology, F-33883, Villenave d’Ornon, France; 4 Cold Spring Harbor Laboratory, 1 Bungtown Road, Cold Spring Harbor, NY 11724, USA; 5 Laboratory of Plant Genetics and Breeding, Department of Biological Production, Faculty of Bioresource Sciences, Kaidobata-nishi 241-438, Shimo-Shinjyo Nakano, Akita City 010-0195, Japan; 6 Metanomics GmbH, Tegeler Weg 33, Berlin, 10589, Germany

**Keywords:** *Arabidopsis thaliana*, *Ramosa3*, Trehalase, Trehalose, *Zea mays*

## Abstract

**Background:**

Trehalose is a non-reducing disaccharide that is used as an osmolyte, transport sugar, carbon reserve and stress protectant in a wide range of organisms. In plants, trehalose 6-phosphate (Tre6P), the intermediate of trehalose biosynthesis, is thought to be a signal of sucrose status. Trehalose itself may play a role in pathogenic and symbiotic plant-microbe interactions, in responses to abiotic stress and in developmental signalling, but its precise functions are unknown. A major obstacle to investigating its function is the technical difficulty of measuring the very low levels of trehalose usually found in plant tissues, as most of the established trehalose assays lack sufficient specificity and/or sensitivity.

**Results:**

A kinetic assay for trehalose was established using recombinant *Escherichia coli* cytoplasmic trehalase (treF), which was shown to be highly specific for trehalose. Hydrolysis of trehalose to glucose is monitored fluorometrically and the trehalose content of the tissue extract is determined from an internal calibration curve. The assay is linear for 0.2-40 pmol trehalose, and recoveries of trehalose were ≥88%. *A*. *thaliana* Col-0 rosettes contain about 20–30 nmol g^-1^FW of trehalose, increasing to about 50–60 nmol g^-1^FW in plants grown at 8°C. Trehalose is not correlated with sucrose content, whereas a strong correlation between Tre6P and sucrose was confirmed. The trehalose contents of ear inflorescence primordia from the maize *ramosa3* mutant and wild type plants were 6.6±2.6 nmol g^-1^FW and 19.0±12.7 nmol g^-1^FW, respectively. The trehalose:Tre6P ratios in the *ramosa3* and wild-type primordia were 2.43±0.85 and 6.16±3.45, respectively.

**Conclusion:**

The fluorometric assay is highly specific for trehalose and sensitive enough to measure the trehalose content of very small amounts of plant tissue. Chilling induced a 2-fold accumulation of trehalose in *A*. *thaliana* rosettes, but the levels were too low to make a substantial quantitative contribution to osmoregulation. Trehalose is unlikely to function as a signal of sucrose status. The abnormal inflorescence branching phenotype of the maize *ramosa3* mutant might be linked to a decrease in trehalose levels in the inflorescence primordia or a downward shift in the trehalose:Tre6P ratio.

## Background

Trehalose (α-d-glucopyranosyl-(1→1)-α-d-glucopyranoside) is a non-reducing disaccharide that is used as an osmolyte, storage reserve, transport sugar and stress protectant by many groups of organisms including bacteria, archaea, fungi and invertebrates. For many years it has been known that trehalose also occurs in non-vascular plants – algae, mosses and liverworts – and primitive vascular plants [1-4]. Indeed one of the earliest reports of trehalose was from the desiccation-tolerant lycophyte *Selaginella lepidophylla*[5]. Among the angiosperms, a small number of desiccation-tolerant resurrection plants, e.g. *Myrothamnus flabellifolia* and *Sporobolus spp.*, accumulate considerable amounts of trehalose under drought conditions, reaching up to 20% of dry weight [6], which may help to maintain cell viability by various mechanisms [7-9]. With the exception of these specialised resurrection plants, it was commonly thought that other flowering plants lacked the capacity to synthesise trehalose, having been displaced by another non-reducing disaccharide – sucrose [10]. The trace amounts of trehalose occasionally reported in some species were generally considered to have a fungal or bacterial origin [11]. This view was completely overturned in 1998 by the unexpected discovery of genes encoding enzymes of trehalose biosynthesis in a desiccation-intolerant model species, *Arabidopsis thaliana*[12,13].

The most common route for trehalose biosynthesis in prokaryotes, and the only one found in eukaryotes [14], is a two-step pathway involving the synthesis of a phosphorylated intermediate, trehalose-6-phosphate (Tre6P). Tre6P is synthesised from UDPglucose and glucose 6-phosphate by trehalose-phosphate synthase (TPS; EC2.4.1.15) and then the phosphate group is hydrolytically removed by trehalose-phosphate phosphatase (TPP; EC3.1.3.12) to yield free trehalose [15]. Blázquez et al. 1998 [12] reported the discovery of the *AtTPS1* gene in *A. thaliana*, which encodes a catalytically active TPS enzyme, while Vogel et al. 2001 [13] reported the presence of the *AtTPPA* and *AtTPPB* genes encoding functional TPPs. Sequencing of the *A. thaliana* genome revealed a total of 11 *TPS* and 10 *TPP* genes in this species [16]. Based on yeast mutant complementation and *in vitro* analysis, *At*TPS1 is the only TPS isoform that has been unequivocally shown to have TPS activity, while all ten isoforms of TPP are catalytically active [17,18]. The functions of the non-catalytic TPS isoforms (*At*TPS2-*At*TPS11) remain unclear. Trehalose is catabolised in plants by trehalase (EC3.2.1.28), which in *A. thaliana* is encoded by a single gene (*AtTRE1*). The enzyme is bound to the plasmalemma with the catalytic site facing the apoplast [3,19]. *TPS*, *TPP* and *TRE* genes have subsequently been identified in all major groups of plants, including both monocots and eudicots among the flowering plants, suggesting that the capacity to synthesise and degrade trehalose is universal in the plant kingdom [14,20].

In *A. thaliana*, *tps1* null mutants are non-viable due to arrest of embryo development at the torpedo stage [21]. The *tps1* embryos can be rescued by dexamethasone-inducible (*GVG::TPS1*) or embryo-specific (*ABI3::TPS1*) expression of TPS1 but, after germination, plants that no longer express the *TPS1* gene grow poorly and flower late or not at all [22,23]. While these findings showed that trehalose metabolism is essential for normal growth and development at all stages of the plant’s life cycle – embryogenesis, vegetative growth and flowering – they did not reveal its function. Many of the growth and developmental defects in the *tps1* and other mutants with altered trehalose metabolism have been ascribed to changes in Tre6P rather than trehalose itself [24]. Consequently, much current research into plant trehalose metabolism is focussed on understanding the precise functions of Tre6P and the molecular mechanisms underlying its regulation of metabolism, growth and development [25-31]. Tre6P can be measured in the pico/femtomole range by high performance anion-exchange liquid chromatography coupled to tandem mass spectrometry (LC-MS/MS) [32]. It was shown that the level of Tre6P increased up to 40-fold when sucrose was supplied exogenously to C-starved *A. thaliana* seedlings, and that Tre6P also changes in parallel with endogenous changes in the level of sucrose during the diurnal cycle. The strong correlation between sucrose and Tre6P led to the proposal that Tre6P acts as a signal of sucrose status [32].

The evidence that Tre6P has an essential signalling function in plants is compelling, but does not exclude a significant role for trehalose itself. In fact, several studies have implicated trehalose as an important factor in plant-microbe interactions and in responses to abiotic stresses (reviewed in [33]). Several studies have suggested a role for trehalose in pathogenic plant-microbe interactions. *Pseudomonas aeruginosa* strain PA14 is a multi-host pathogen that infects nematodes, insects vertebrates and plants. Mutants of *P. aeruginosa* that lack the capacity to synthesise trehalose are unable to infect *A. thaliana*, but unaffected in their ability to infect non-plant hosts, indicating that trehalose is a virulence factor in *P. aeruginosa* that is specific for plant infection [34]. Although its precise role in this plant-pathogen interaction is not yet established, trehalose synthesis appears to be necessary for the bacterium to take up nitrogen-containing nutrients from the plant and to replicate in the extracellular spaces of the plant. Similarly, *Magnaporthe grisea*, a filamentous fungal pathogen that causes blast disease in rice, requires a functional *Tps1* gene in order to infect its host plant [35]. Most *A. thaliana* accessions are highly susceptible to infection by the clubroot pathogen – *Plasmodiophora brassicae* – but Burren-0 (Bur-0) shows partial resistance that has been linked to its greater tolerance of pathogen-induced trehalose accumulation [36]. Infection of *A. thaliana* with this pathogen had previously been shown to induce trehalase expression in the plant [37]. Supplying trehalose exogenously to *A. thaliana* induced a number of pathogen defence related genes, as well as genes linked to abiotic stress responses [38,39], and trehalose application to wheat (*Triticum aestivum*) induced partial protection against the fungal pathogen *Blumeria graminis* (powdery mildew) [40,41].

There is evidence that trehalose also plays a role in some beneficial plant-microbe interactions, including rhizobial symbioses in legumes [42-48] and ectomycorrhizal symbioses with tree species [49-51]. The non-symbiotic association of a plant growth-promoting rhizobacterium, *Burkholderia phytofirmans*, with grapevine led to activation of trehalose metabolism and improved chilling tolerance in the plants [52]. Inoculation of maize with another rhizobacterium, *Azospirillum brasiliense*, that had been engineered to increase trehalose production, led to a 73% increase in plant biomass, and improved the plants’ drought tolerance and grain yield [53].

In addition to the trehalose-accumulating resurrection plants, other species show changes in their trehalose metabolism in response to abiotic stresses. In *A. thaliana*, the expression of many of the genes associated with trehalose biosynthesis is altered in response to cold, osmotic and salt stresses [54]. In rice, *OsTPP1* transcripts were highly induced within a few hours of chilling stress and trehalose levels increased, suggesting a possible role for trehalose in the plants’ adaptation to cold [55]. Over-expression of *OsTPP1* in transgenic rice plants was found to enhance their tolerance of cold and salt stresses, lending support to this hypothesis [56]. Expression of *OsTPP1* (and *OsTPP2*) was also increased in transgenic rice plants that over-express *Os*MYBS3, a cold-induced transcription factor that is required for chilling-induced cold tolerance in rice [57]. From these results, it has been suggested that cold-induced changes in *OsTPP* expression and trehalose content in wild-type plants may be a transient response that helps to protect the plant in the short-term while it adapts to the cold, and may act as a signal for triggering long-term adaptive responses.

Expression of heterologous *TPS* and *TPP* genes (from yeast or bacteria) in plants has been reported to improve tolerance to drought, salt, cold and oxidative stresses [58-61]. However, the plants often showed pleiotropic effects, such as changes in leaf shape and senescence, that could be ascribed to changes in the level of Tre6P [24,62]. Improvements in abiotic stress tolerance without obvious pleiotropic effects have been reported from: (i) expression of bifunctional TPS-TPP enzymes [63-65]; (ii) targeting of the heterologous enzyme(s) to the chloroplasts [66]; and (iii) expression of *TPS* and *TPP* genes under the control of stress-inducible promoters [67,68].

Interestingly, in many of these studies, the levels of trehalose were below the limits of detection of the assay methods employed, not only in the wild-type control plants but also in the stress-tolerant transgenic lines, unless the plants were treated with validamycin A to inhibit the endogenous trehalase [58,69]. Therefore, it is doubtful that trehalose made a quantitatively important contribution to osmoregulation in these plants, or exerted protection via mass action effects, unless its distribution was highly localised.

The *A. thaliana tre1-1* null mutant has no detectable trehalase activity and accumulates about 4-fold more trehalose than wild-type plants. Surprisingly, despite its higher trehalose content, the *tre1-1* mutant was found to be more susceptible to drought stress than the wild-type [70]. Drought stress leads to an increase in abscisic acid (ABA) levels in plants, triggering closure of the stomata and so reducing water loss from the leaves, but this response to ABA was impaired in the *tre1-1* mutant. In contrast, in *AtTRE1* over-expressing lines, which had less trehalose than wild type plants, the stomata were hypersensitive to ABA and the plants showed greater drought tolerance than the wild-type [70].

These studies of mutants and transgenic lines have shown that the effects of manipulating trehalose metabolism on abiotic stress tolerance are complex, and probably do not involve trehalose acting as a compatible solute and stress protectant in the classical sense [71]. Together with the transcript profiling studies that showed trehalose-induced changes in expression of biotic and abiotic stress related genes [38,39,72,73], they point to trehalose acting as a signal molecule.

The possibility that both Tre6P and trehalose have signalling functions in plants complicates phenotypic analysis and interpretation of plants with altered TPS and/or TPP activities. A good example is the maize *ramosa3* (*ra3*) mutant, which has abnormally branched male and female inflorescences. This phenotype was shown to be caused by a lesion in a *TPP* gene (*RAMOSA3*) that is expressed in discrete domains subtending the axillary inflorescence meristems [74], although the underlying molecular mechanism is still unresolved [29]. Conceptually, the developmental defect in the mutant could result from an increase in Tre6P or a decrease in trehalose due to lower TPP activity in the affected cells, or a combination of both changes (i.e. a shift in the trehalose:Tre6P ratio). It is also possible that the phenotype is independent of any effect on trehalose metabolism and linked to some non-catalytic property of the RAMOSA3 protein instead. To distinguish between these possibilities, there is an obvious need to measure both Tre6P and trehalose in developing inflorescence primordia from wild type and mutant plants.

As noted above, Tre6P can be measured in the pico/femtomole range by LC-MS/MS [32], but few of the established trehalose assay methods are sensitive and specific enough to measure trehalose in small amounts of plant tissue. Trehalose has been measured in yeast cells by colorimetric detection using anthrone, but this reagent is not specific enough for use with plant extracts as it gives a strong colour reaction with sucrose [75,76]. Paper chromatography and crystallization of trehalose [77] is both time consuming and insensitive, requiring large amounts of plant material and multiple fractionation steps, and was found to be unreliable as a quantitative method [76,78]. Gas liquid chromatography [79] and ^13^C-nuclear magnetic resonance [80] are more quantitative methods, but lack sensitivity and also require expensive equipment. High-performance anion-exchange chromatography with pulsed amperometric detection (HPAEC-PAD) offers greater sensitivity, with a detection limit in the range of about 15–300 nmol [81,82]. However, this method was shown to be unsuitable for measurement of trehalose in *A. thaliana*, flax (*Linum usitatissimum*) and sugar beet (*Beta vulgaris*) extracts, due to coelution of trehalose with other compounds, which were not identified, resulting in a 7- to 13-fold over-estimation of trehalose [83]. Gas chromatography coupled to detection by mass spectrometry (GC-MS) provides both high sensitivity and specificity [83,84], but requires expensive equipment that is not available in many laboratories and has a relatively low throughput. Enzymatic assays, based on hydrolysis of trehalose by trehalase and quantification of the resulting glucose [85], potentially offer good specificity and high throughput, but the colorimetric or spectrophotometric methods commonly used to measure glucose are usually not sensitive enough for trehalose determination in plant extracts.

**Figure 1:**
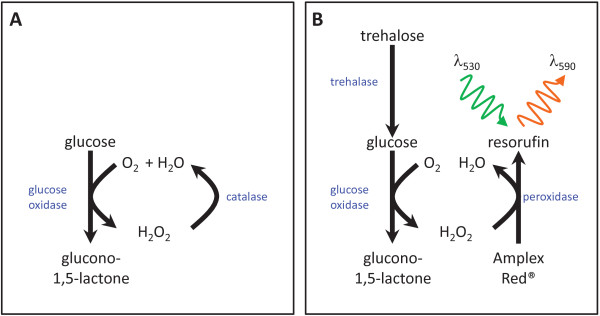
**Principle of the fluorometric assay of trehalose.** (**A**) First, glucose is eliminated from the tissue extract by incubation with glucose oxidase and catalase. (**B**) After inactivation of the glucose oxidase and catalase, trehalose is then hydrolysed to glucose by trehalase. Glucose is determined by coupling to peroxidation of a fluorogenic substrate, Amplex Red^®^, using glucose oxidase and peroxidase and measuring the increase in fluorescence at 590 nm.

Here we describe an enzymatic assay for trehalose that is both highly specific and sensitive enough to measure trehalose in as little as 1 mg fresh weight of plant tissue. The assay is based on specific hydrolysis of trehalose to glucose by recombinant *E. coli* cytoplasmic trehalase (treF), coupled to fluorometric detection of the glucose by glucose oxidase and peroxidase (Figure 1). Using this assay we were able to quantify trehalose in inflorescence primordia from the maize *ramosa3* mutant and wild type plants, and compare these with the levels of Tre6P measured by LC-MS/MS in the same extracts. We also investigated whether trehalose, like Tre6P, changes in parallel with sucrose during the diurnal cycle in *A. thaliana* rosettes, and studied the effect of low temperature on the trehalose levels in this species.

## Results and discussion

### Substrate specificity of trehalases from porcine kidney and *E. coli*

**Figure 2:**
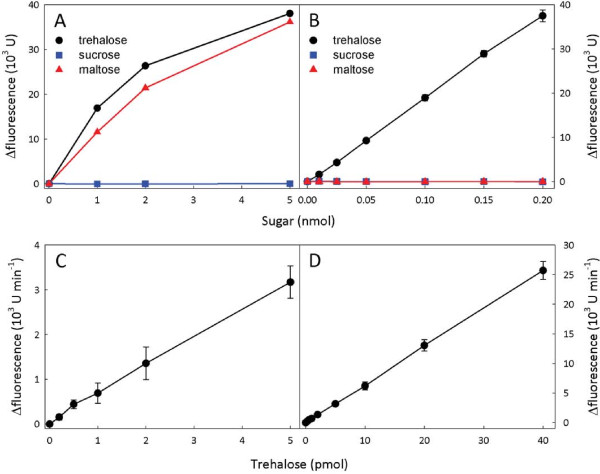
**Specificity of different trehalases and linearity of the fluorometric assay of trehalose.** Hydrolysis of trehalose and other disaccharides by (**A**) porcine kidney trehalase and (**B**) *E. coli* cytoplasmic trehalase (treF). The amount of glucose released after addition of trehalase was determined in an end-point assay using glucose oxidase, peroxidase and Amplex Red^®^. The increase in fluorescence is expressed in arbitrary fluorescence units. The linearity of the optimised kinetic assay for trehalose using *E. coli* cytoplasmic trehalase was tested with 0–5 pmol trehalose (**C**) and 0–40 pmol trehalose (**D**). The initial rate of the reaction was monitored fluorometrically (arbitrary fluorescence units min^-1^). Data are mean ± SD (*n* = 2 or 3).

Preliminary experiments were carried out using a commercially available porcine kidney trehalase (Sigma-Aldrich) to test the specificity of this enzyme. Glucose production was determined fluorometrically by coupling the oxidation of glucose to peroxidation of the fluorogenic substrate 10-acetyl-3,7-dihydroxyphenoxazine (Amplex Red^®^), using glucose oxidase and peroxidase (Figure 1B). Reaction mixtures initially contained known amounts of various glucose-containing sugars, the coupling enzymes and Amplex Red^®^. When the baseline fluorescence signal was stable, porcine kidney trehalase was added and the production of glucose was continuously monitored until the reaction was complete. The difference in fluorescence (Δfluorescence) between the baseline and the end point of the reaction was calculated and the results are shown in Figure 2A. The porcine kidney trehalase was found to hydrolyse not only trehalose but also maltose, yielding similar amounts of glucose with both sugars. There was no apparent reaction with sucrose. The maltose content of wild-type *A. thaliana* leaves ranges from about 5 nmol g^-1^FW during the day up to 90 nmol g^-1^FW at night [86,87], and so is similar or higher than the level of trehalose measured by GC-MS in this tissue – about 50 nmol g^-1^DW (equivalent to approximately 5 nmol g^-1^FW) [83]. Thus the level of maltose in plant extracts is sufficient to seriously interfere with any assay of trehalose content using the porcine kidney trehalase, which does not distinguish between these two sugars. Therefore, we sought an alternative form of trehalase that would be specific for trehalose.

The *Escherichia coli* cytoplasmic trehalase (treF) was reported to be highly specific for trehalose, showing no hydrolytic activity with maltose, sucrose or lactose [88], suggesting that it might be suitable for enzymatic assay of trehalose in plant tissues. The treF trehalase was over-expressed in *E. coli* as a His_6_-tagged fusion protein, and purified to near homogeneity by immobilised metal affinity chromatography, gel filtration and anion-exchange chromatography (see Additional file 1: Figure S1). End point assays with glucose-containing oligosaccharides that are likely to be present in plant extracts confirmed that the treF enzyme has no significant hydrolytic activity with maltose, sucrose, maltotriose or cellobiose (Figure 2B and Additional file 2: Figure S2). The amount of glucose released from trehalose was directly proportional to the amount of trehalose in the assay (Figure 2B). Based on these results, the treF trehalase was judged to be suitable for determination of trehalose in plant extracts.

### Optimisation and validation of a kinetic assay for trehalose

Plant tissues usually contain substantial amounts of glucose, for example, *A. thaliana* leaves typically have around 0.5-5 μmol g^-1^FW [89]. This will obviously interfere with any trehalose assay based on measurement of the glucose, unless the original glucose in the extract is first removed. This can be achieved by incubating the tissue extract with glucose oxidase and catalase to bring about quantitative oxidation of glucose to gluconate. The destruction of the reaction by-product, hydrogen peroxide, by catalase regenerates the oxygen needed for oxidation of glucose and drives the reaction to completion (Figure 1A). After removal of glucose, the enzymes are inactivated by heating. Trehalose can then be determined by addition of trehalase and measurement of glucose production using glucose oxidase, peroxidase and Amplex Red^®^ (Figure 1B). With trehalose standards, the end-point assay described in the previous section was shown to give a linear response for 10–200 nmol of trehalose (Figure 2B). As it was desirable to measure trehalose in the same extracts as those used for measurement of Tre6P by LC-MS/MS, the following experiments were performed using chloroform-methanol extracts prepared as described in [32]. Initial trials with extracts from various *A. thaliana* tissues indicated the end-point method was sensitive enough to measure trehalose in flowers, which have relatively high levels of trehalose (218 ± 47 nmol g^-1^FW), but the levels of trehalose in leaves (28 ± 6 nmol g^-1^FW) and roots (25 ± 5 nmol g^-1^FW) were much lower and close to the limit of detection of the end-point assay. Therefore, a potentially more sensitive kinetic assay was tested.

The kinetic assay is based on measurement of initial rates of trehalase activity with sub-saturating amounts of trehalose, below the *K*_*m*_ (1.9 mM) of the *E. coli* treF trehalase [88], and limiting amounts of trehalase in the assay. Under such conditions, the activity of the enzyme is essentially linear with respect to the concentration of trehalose. To calibrate the assay, it is necessary to generate an internal standard curve for each sample. This is done by addition of known amounts of trehalose (or water for the blank) to equal aliquots of the tissue extract, from which all traces of trehalose and glucose have previously been removed by prior incubation with trehalase (treF), glucose oxidase and catalase. An equivalent aliquot of the extract is incubated with glucose oxidase and catalase to remove only glucose. Trehalose is then assayed in all of the aliquots in parallel by addition of a limiting amount of treF trehalase, along with glucose oxidase, peroxidase and Amplex Red^®^. The initial rate of each reaction is monitored fluorometrically. The slope of the linear portion of each reaction is calculated and the blank rate is subtracted. The amount of trehalose in the original extract is determined by comparing the rate of the reaction for the aliquot that was not pre-incubated with trehalase with the rates of the reactions spiked with known amounts of trehalose. All reactions were carried out (in duplicate) in 96-well microplates, allowing several samples to be analysed simultaneously. Measurements were done on a microplate reader operating in fluorescence mode, using an excitation wavelength of 530 nm and detection of emission at 590 nm. Depending on the expected amount of trehalose in the extract, a low (0.2-4 pmol) or high (2–20 pmol) range of trehalose standards was used for the internal calibration curve. Calibration plots were highly linear up to 40 pmol of trehalose (Figure 2C-D), although the slope can vary considerably between tissue types, presumably due to differences in the degree of quenching by other compounds in the extract. However, the use of an internal calibration curve for each sample automatically compensates for any variation in quenching or background fluorescence from components of the plant extract.

We also compared the sensitivity and linearity of the assay using Amplex Red^®^ and an alternative formulation of the fluorogenic substrate, Amplex UltraRed^®^ (Invitrogen), which is claimed by the manufacturer to give a higher fluorescence yield. In trials with trehalose standards and *A. thaliana* leaf extracts, Amplex UltraRed^®^ gave a marginally higher fluorescence signal than with Amplex Red^®^, but slightly higher blanks (data not shown). Therefore, there appeared to be no great advantage in using Amplex UltraRed^®^, and the original Amplex Red^®^ reagent was used in most of the subsequent analyses.

Experiments were carried out to determine the recovery of trehalose from extraction of *A. thaliana* leaf samples with chloroform-methanol. Quadruplicate aliquots of frozen tissue powder (12–15 mg FW, containing 270–580 pmol of trehalose) were spiked with either 500 or 1500 pmol of trehalose before extraction. Spiked and non-spiked control samples were extracted with chloroform-methanol [32], and trehalose was measured in 5 μl of the extract (total extract volume 250 μl). The non spiked samples contained 30.3 ± 7.5 nmol g^-1^FW of trehalose (mean ± SD, *n*=4). From the amounts of trehalose measured in the spiked samples, the recoveries of the added 500 and 1500 pmol of trehalose were calculated to be 98 ± 22% and 92 ± 16%, respectively. In a similar experiment using Amplex UltraRed^®^ reagent, non-spiked samples contained 32.8 ± 6.0 nmol g^-1^FW (mean ± SD, *n* = 4), and the recoveries of 500 and 1500 pmol of trehalose were 105 ± 10% and 88 ± 4%, respectively. These experiments demonstrated the reliability of the chloroform-methanol extraction method for quantitative recovery of trehalose, and that there was no significant loss of trehalose during the initial incubation with glucose oxidase/catalase to remove glucose.

### Diurnal changes in the trehalose content of *A. thaliana* leaves and its response to low temperature

**Figure 3:**
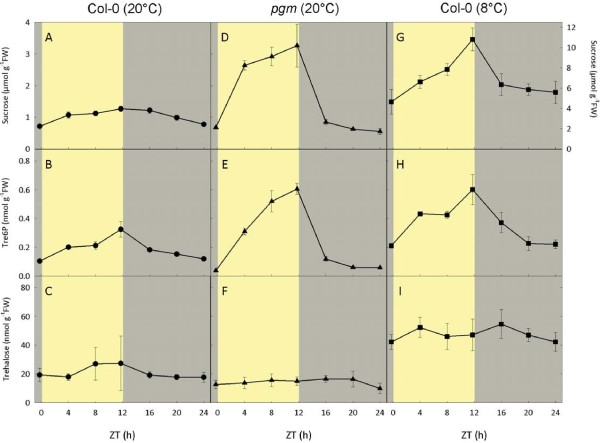
**Diurnal changes in sucrose, Tre6P and trehalose content of *Arabidopsis thaliana *rosettes.** (**A**-**C**) Wild-type *A. thaliana* Col-0 plants and (**D**-**F**) plastidial phosphoglucomutase (*pgm*) mutant plants were grown in a 12 h light/12 h dark diurnal cycle at 20°C. (**G**-**I**) Wild type Col-0 plants were also grown at 20°C for 3 weeks and then transferred to 8°C for 1 week. Rosettes were harvested from 4-week-old plants at 4-h intervals through a complete 24-h diurnal cycle for measurement of trehalose and other metabolites. n.b. expanded *y*-axis scale for sucrose content of Col-0 plants at 8°C (**G**). Data are mean ± SD, *n* = 4 (WT, 20°C) or 5 (WT, 8°C and *pgm*).

The amount of Tre6P in *A. thaliana* seedlings and soil-grown plants was found to be correlated with the level of sucrose [32]. As Tre6P is the direct precursor of trehalose, it might be expected that trehalose also changes in response to diurnal fluctuations in sucrose. To investigate this possibility, trehalose, Tre6P and sucrose were measured in rosettes of wild type *A. thaliana* Col-0 plants grown at 20°C and harvested at 4-h intervals through a 12 h light/12 h dark diurnal cycle. All three metabolites showed a moderate increase during the day, before falling back down at night (Figure 3A-C).

The starch-deficient plastidial phosphoglucomutase (*pgm*) mutant shows more pronounced diurnal changes in sugars, therefore, we carried out a similar analysis of *pgm* plants grown under the same conditions. The *pgm* plants accumulated about 3-fold higher levels of sucrose by the end of the day (Figure 3D) than wild-type Col-0 plants. There was a sharp drop in sucrose levels by 4 h into the night, and sucrose decreased even further by the end of the night, falling below the levels seen in the wild-type plants (Figure 3A). These marked changes in sucrose content were closely paralleled by a large diurnal fluctuation in the level of Tre6P in the *pgm* plants (Figure 3E). In contrast, trehalose levels remained almost constant throughout the light–dark cycle (Figure 3F), and were slightly lower than those seen in the wild-type Col-0 plants (Figure 3C).

Plants exposed to low temperatures often accumulate substantial amounts of sucrose and other sugars [90]. Therefore, it was of interest to determine whether low temperatures affect the level of trehalose in *A. thaliana*. Wild type plants were grown in a 12 h/12 h light–dark cycle as above for 3 weeks and then exposed to a chilling temperature of 8°C for 1 week. These plants contained about 6–10 times higher levels of sucrose throughout the diurnal cycle than those grown at 20°C, peaking at the end of the day and then falling at night (Figure 3G). As seen in the other plants, Tre6P showed a similar diurnal rhythm to sucrose, but the levels were about 2-fold higher than those seen in non-chilled wild-type plants (Figure 3H). Trehalose was about 2.5-fold higher in the cold-treated plants than those grown at 20°C, but again showed only minor fluctuations during the diurnal cycle (Figure 3I).

**Figure 4:**
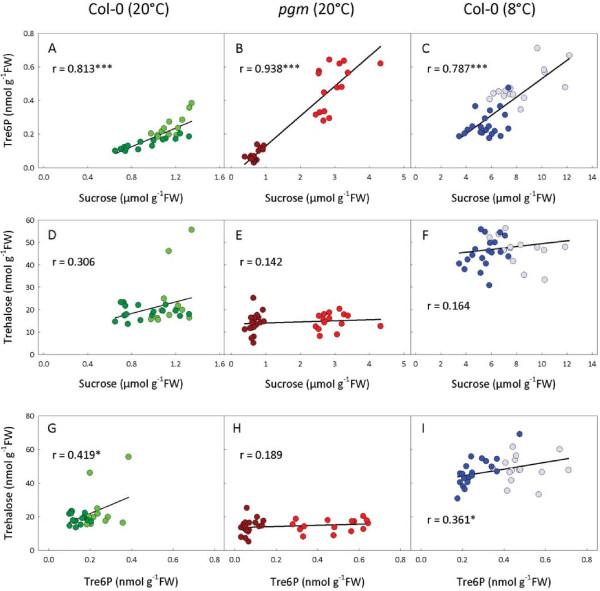
**Relationships between trehalose, Tre6P and sucrose content of *Arabidopsis thaliana *rosettes.** Rosettes were harvested from 4-week-old plants at 4-h intervals through a 24-h diurnal cycle for metabolite analysis: (**A,D,G**) wild-type Col-0 grown at 20°C, (**B,E,H**) *pgm* mutant grown at 20°C, and (**C,F,I**) wild-type Col-0 grown at 20°C for 3 weeks and at 8°C for 1 week (see Figure 3). The plots show pairwise comparisons of metabolite data from individual samples. Dark symbols represent samples harvested at night, pale symbols those harvested in the light. The Pearson correlation coefficient (r) was calculated for each pair of metabolites using SigmaPlot 11 (Systat Software, Inc., Chicago, IL, USA), and significant *P* values are indicated by asterisks: *P<0.05, **P<0.01, ***P<0.001.

To quantify the relationships between sucrose, trehalose and Tre6P, pairwise comparisons of metabolite levels were plotted (using data from the individual samples), and the Pearson’s correlation coefficient (r) calculated for each pair of metabolites in the three experiments (Figure 4). In all three sets of plants, Tre6P was strongly correlated with sucrose, with r-values ranging from 0.787 (Col-0 at 8°C) to 0.938 (*pgm* at 20°C), and the correlations were highly significant, with *P* values ranging from 1.5×10^-7^ to 9.1×10^-17^ (Figure 4A-C). In contrast, there was no significant correlation between trehalose and sucrose (Figure 4D-F). Trehalose was weakly correlated with Tre6P in both sets of wild-type Col-0 plants (*P*<0.05), but not in the *pgm* plants (Figure 4G-I).

These results confirm the strong relationship between sucrose and Tre6P that has previously been observed in various *A. thaliana* tissues [31,32], lending weight to the hypothesis that Tre6P acts as a signal of sucrose status in plants. Interestingly, although Tre6P was highly correlated with sucrose in all three sets of plants, the slope of the regression plot was substantially lower for the cold-treated wild-type Col-0 plants (0.054) than those grown at 20°C (0.277), or the *pgm* plants (0.178). This suggests that while the strong relationship between these metabolites is maintained at low temperature, it is poised at a lower Tre6P:sucrose ratio. This may allow the plants to remain responsive to fluctuations in the level of sucrose even though the absolute amounts of sucrose are much higher than in non-chilled plants. The high concentrations of sucrose in the cells will at least partially offset the thermodynamic decrease in maximal catalytic activities of the enzymes at low temperature, and so help to maintain metabolic fluxes under these conditions. Sucrose accumulation may also be part of the plant’s acclimatory response, offering some protection against anticipated freezing temperatures. Even though the low-temperature treated plants had moderately elevated trehalose levels (about 2.5 times higher than at 20°C), it is worth noting that the absolute amounts of sucrose were over 200-fold higher than those of trehalose. Thus trehalose does not appear to make a substantial quantitative contribution to osmoregulation under these conditions, and any direct protective effect seems doubtful. These results are consistent with the increased but still low absolute amounts of trehalose found in rice and grapevine after chilling [52,55].

The absence of any obvious correlation between sucrose and trehalose (Figure 4D-F) argues against trehalose having a similar sucrose-signalling function to Tre6P. The relationship between Tre6P and trehalose was surprisingly weak, with no significant correlation at all in the *pgm* mutant (Figure 4G-I). The level of trehalose in the plants is presumably governed by: (i) the supply of Tre6P, (ii) the activity of TPP, and (iii) the activity of trehalase. Although experimental data are sparse, it is likely that Tre6P and trehalose are produced mainly in the cytosol [91], whereas the trehalase enzyme has been shown to be anchored in the plasmalemma with the active site facing the apoplast [3,19]. Presumably there must be some kind of transport mechanism for exporting trehalose out of the cell to make it accessible to the trehalase. Although nothing is known about how this is brought about, the transport of trehalose out of the cell could be an additional control point for regulating the level of trehalose. These uncertainties highlight the substantial gaps in our knowledge about the subcellular compartmentation of trehalose metabolism in plant cells.

### Metabolite analysis of inflorescence primordia from wild-type and *ramosa3* maize plants

Previous attempts to measure Tre6P and trehalose in developing inflorescence primordia from the maize *ramosa3* mutant had been unsuccessful, leaving open the question of whether the loss of the RAMOSA3 TPP activity affected either or both of these metabolites [74]. The fluorometric assay we have established is not only sensitive enough to measure trehalose in very small amounts of plant tissue (as little as 1 mg), but also has the advantage that Tre6P can be measured in the same extracts by LC-MS/MS [32]. This opened up the possibility to measure both of these metabolites in *ramosa3* inflorescence primordia.

**Figure 5:**
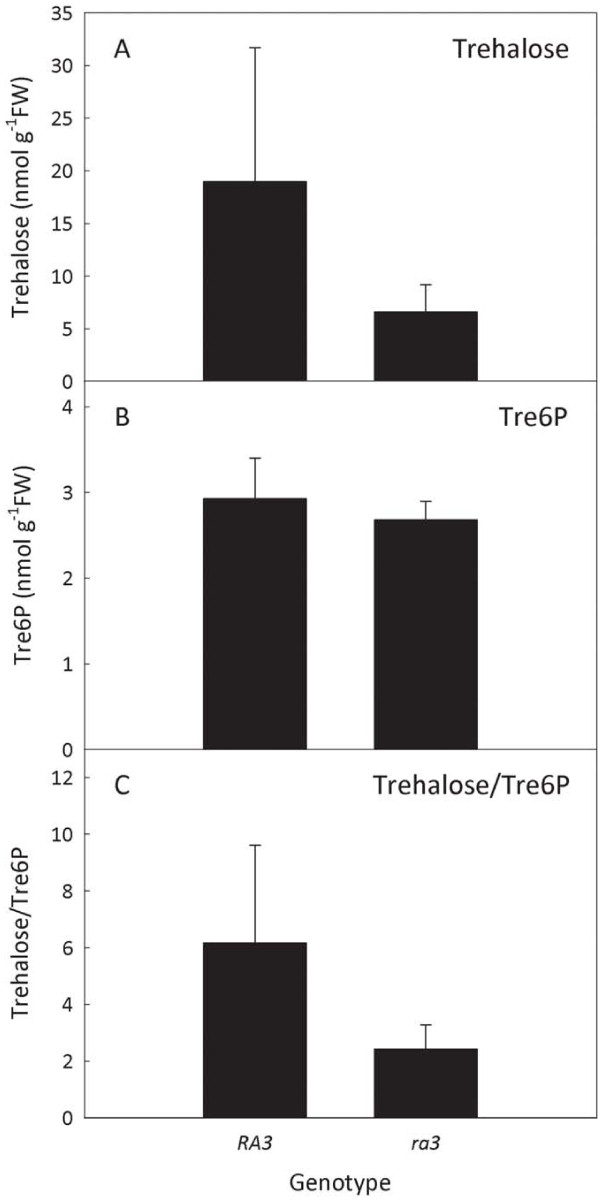
**Trehalose and Tre6P content in maize *ramosa3 *inflorescence primordia.** Ear inflorescence primordia (1-4 mm in length) were harvested from 9 to 10-week-old plants of the *ramosa3* (*ra3*) mutant and wild type B73 (*RA3*) maize plants. Trehalose (**A**) was measured fluorometrically and Tre6P (**B**) was measured by LC-MS/MS. The trehalose:Tre6P ratio (**C**) was calculated for each individual sample. Data are mean ± SD (*n* = 4).

Ear inflorescence primordia were harvested from wild-type (B73) and *ramosa3* maize plants grown in the field, at the 1–4 mm stage when the *RAMOSA3* gene is expressed (from 9 to 10-week-old plants). There appeared to be a tendency for the *ramosa3* primordia to have lower trehalose than those from wild-type plants (Figure 5A), but the large biological variation between samples, particularly those from the wild-type plants, rendered the difference non-significant according to Student’s *t*-test (*P*=0.106). There was no difference in Tre6P levels between wild type and mutant primordia (Figure 5B). It is worth noting that the absolute levels of Tre6P (2.7-2.9 nmol g^-1^FW) are at least 5-fold higher than those in *A. thaliana* leaves (Figure 3D-F), which may reflect the more densely cytoplasmic nature of the cells from developing primordia compared to the highly vacuolated leaf mesophyll cells. Interestingly, however, trehalose levels were not higher on a fresh weight basis than those found in *A. thaliana* rosettes (Figures 3 and 5).

When analysing such small amounts of tissue, the measurement of the sample fresh weight is likely to be a significant source of experimental error. The ratio of trehalose:Tre6P in a given sample is independent of the weight, and so might be a more robust parameter for comparison of mutant and wild-type primordia. The trehalose:Tre6P ratio was calculated for the individual samples and the results are shown in Figure 5C. As observed for the absolute amounts of trehalose, the trehalose:Tre6P ratio showed a strong tendency to be lower in the *ramosa3* primordia than in the wild-type but the difference was not statistically significant (*P*=0.08), although closer to the 0.05 threshold of significance.

It should be noted that the *RAMOSA3* gene is expressed in only a minority of the cells in the inflorescence primordia, even at the peak in its expression level [74]. Therefore, even large changes in trehalose and/or Tre6P levels in the *RAMOSA3*-expressing cells might be partially masked by the pools of these metabolites in the promordia extracts that are derived from cells that do not express *RAMOSA3*. Nevertheless, based on these results, we tentatively conclude that the abnormal branching phenotype of the *ramosa3* inflorescences is not linked to a change in the level of Tre6P in the primordia. The tendency for trehalose to be decreased in the *ramosa3* primordia would seem to be consistent with a loss or reduction in total TPP activity in the affected cells. However, further experiments will be needed to determine if this tendency is mechanistically linked to the aberrant development of the inflorescences. For example, it would be interesting to test whether the mutant can be complemented by a catalytically incapacitated form of the RAMOSA3 TPP. If the mutant is complemented by such a protein, this would suggest that the developmental phenotype is linked to some other property of the RAMOSA3 protein and its TPP activity is coincidental. If the mutant is not complemented, this would suggest that the phenotype really is linked to disruption of trehalose metabolism in the affected cells.

## Conclusions

We have developed and validated a highly specific and sensitive fluorometric assay for trehalose. The level of trehalose in *A. thaliana* rosettes showed no obvious dependence on sucrose content. Therefore, trehalose is unlikely to be involved in sucrose signalling pathways, whereas its precursor, Tre6P, was confirmed to be highly correlated with sucrose, supporting its proposed function as a sucrose signal. Trehalose is weakly correlated with Tre6P but only in wild-type Col-0 plants. Trehalose was moderately elevated in *A. thaliana* Col-0 plants exposed to low temperature (8°C), compared to non-chilled plants (20°C). However, the absolute levels of trehalose were still very low, being several orders of magnitude less than sucrose, and so insufficient to make a quantitatively important contribution to osmoregulation. Ear inflorescence primordia from the maize *ramosa3* mutant contained similar levels of Tre6P to primordia from wild-type plants, indicating that the abnormal branching phenotype of the mutant inflorescences is probably not linked to changes in Tre6P content in the affected primordia cells. There was a tendency for trehalose and the trehalose:Tre6P ratio to be lower in the mutant compared to wild-type, but the differences were not statistically significant. This leaves open the question of whether the phenotype is linked to disturbance of trehalose metabolism or to some non-catalytic function of the RAMOSA3 protein. Although the function of the RAMOSA3 protein could not be clearly resolved from the metabolite data, the fact that we were able to measure both trehalose and Tre6P in such small amounts of tissue, where previous attempts had failed, demonstrates the potential of the newly established assay method to advance our understanding of trehalose metabolism and its functions in plants.

## Methods

### Plant material

*Arabidopsis thaliana* (L.) Heynh. accession Col-0 wild type and *pgm* mutant [92] plants were grown in soil with a 12 h light/12 h dark diurnal cycle, an irradiance of 130–160 μE m^-2^s^-1^ and a constant temperature of 20°C, unless stated otherwise. Rosettes were harvested from 25-day-old plants and immediately frozen in liquid nitrogen under ambient irradiance. The frozen plant tissue was ground to a fine powder at liquid nitrogen temperature using a ball mill and stored at −80°C until analysis. Maize (*Zea mays*) cv. B73 (wild type) and *ramosa3* mutant plants that had been introgressed about six times into B73 [74] were grown in the field at the Cold Spring Harbor Laboratory field station, NY, USA, in May-August 2007. Ear inflorescence primordia (1–4 mm in length) were rapidly dissected from 9 to 10-week-old plants in the field and immediately frozen in liquid nitrogen.

### Reagents

Recombinant glucose oxidase from *Aspergillus niger* (specific acivity ≥167 U mg^-1^ protein, containing ≤10 U mg^-1^ protein of catalase) was obtained from Sigma-Aldrich (Taufkirchen, Germany). Peroxidase from horseradish (Grade I; 250 U mg^-1^ lyophilisate, containing <0.7% catalase) was obtained from Roche (Mannheim, Germany). Porcine kidney trehalase (≥1.0 U mg^-1^ protein) was obtained from Sigma-Aldrich. Amplex Red^®^ (10-acetyl-3,7-dihydroxyphenoxazine) and Amplex RedUltra^®^ were purchased from Molecular Probes Europe (Leiden, The Netherlands).

### Expression and purification of recombinant *E. coli* treF trehalase

The *E. coli treF* coding region encoding cytoplasmic trehalase (EC 3.2.1.28) was amplified from *E. coli* genomic DNA by PCR and cloned between the NcoI and EcoRI sites of expression plasmid pETM11. The treF protein was expressed as an N-terminal His_6_-tagged fusion protein in *E. coli* strain Rosetta™ (EMD Millipore, Billerica, MA, USA). Protein expression was induced at a cell density of approx. 4×10^8^ cells mL^-1^ (OD_600_ = 0.5) with 0.5 mM isopropyl β-D-thiogalactoside. The induced cells were incubated at 20°C for 16 h, harvested by centrifugation at 5000 × *g* (4°C) for 10 min, suspended in extraction buffer (50 mM Hepes-Na^+^, 300 mM NaCl, pH 7.5,) containing 1 mM phenylmethylsulfonyl fluoride, and extracted by single passage through an EmulsiFlex^®^-C3 high pressure homogenizer (Avestin Inc., Ottawa, Canada) at 110–120 MPa peak pressure. The cell lysate was clarified by centrifugation at 20,000 × *g* (4°C) for 10 min, and the supernatant comprised the soluble extract. All subsequent procedures were carried out at 4°C. The His_6_-treF protein was purified by immobilized metal affinity chromatography on Talon™ Co^2+^ resin (Clontech Laboratories, Inc., Mountain View, CA, U.S.A.) according to the manufacturer’s instructions. The enzyme was further purified by size-exclusion chromatography on a HiLoad16/60 Superdex 200 column (Amersham Biosciences Europe GmbH, Freiburg, Germany), equilibrated with 50 mM Hepes-Na^+^, 5 mM MgCl_2_, 0.5 mM EDTA, pH 7.5. The flow rate was 1.5 ml min^−1^, and 2-ml fractions were collected. Fractions from the major protein peak were pooled and applied to an HR 5/5 MonoQ^®^anion-exchange column (Amersham Biosciences), equilibrated with 50 mM Hepes-Na^+^, 5 mM MgCl_2_, 0.5 mM EDTA, pH 7.5. His_6_-treF was eluted with a linear gradient of 0–0.5 M NaCl (30 ml) in the same buffer. The flow rate was 1 ml min^−1^ and 0.5-ml fractions were collected. Aliquots (2 μl) of the major protein peak fractions were analysed by SDS-polyacrylamide gel electrophoresis on a 10% polyacrylamide gel stained with Coomassie Blue R-250. Fractions containing a single 67-kDa protein band were pooled and concentrated to a volume of approx. 200 μl using a Centricon Plus-20 (30 kDa cut-off) centrifugal concentrator (EMD Millipore). The purified His_6_-treF had a specific activity of 80 μmol min^-1^ mg^-1^protein (1333 nkat mg^-1^protein). The enzyme was stored in the MonoQ column elution buffer at −80°C and was stable for at least 2 years.

### Trehalose determination

Trehalose was extracted from frozen plant tissue using chloroform-methanol as described in Lunn et al. 2006 [32]. The trehalose content of plant extracts was determined using a kinetic assay, measuring trehalase activity in the presence of limiting concentrations of trehalose. Trehalase activity was monitored fluorometrically by coupling with glucose oxidase and peroxidase. Six aliquots (5 μl) of the tissue extract (each corresponding to approx. 50 μg FW of tissue) were placed in separate wells of a Costar^®^ black polystyrene 96-well microplate (Corning Incorporated, NY, USA) – one aliquot for trehalose determination and the other five for generation of an internal calibration curve including a blank. A reaction mixture (35 μl) containing: 4 μl of 10× reaction buffer (500 mM KH_2_PO_4_-KOH, 10 mM MgCl_2_, 100 mM NaCl, pH 7.5), 100 nkat catalase and 67 nkat glucose oxidase was added to each aliquot. Five of the reaction mixtures were supplemented with 0.27 nkat trehalase (treF). The microplate was loosely covered and incubated at 30°C with constant shaking for 60 min to remove glucose, or to remove both glucose and trehalose in the five reactions containing trehalase. The microplate was sealed with adhesive foil and heated at 80°C for 15 min to inactivate the enzymes. After cooling, the plate was centrifuged at 1000×*g* for 1 min and placed on ice.

Water (5 μl) was added to the aliquot that had been incubated without trehalase. To generate an internal calibration curve, 5 μl of water were also added to one of the five trehalase-treated samples as a blank, and the remaining four trehalase-treated aliquots were supplemented with 5 μl of standard solutions containing 0.04-0.8 μM trehalose (i.e. 1–4 pmol) or 5 μl of 1.0-4.0 μM trehalose (i.e. 5–20 pmol). To each of the six wells was then added 55 μl of a reaction mixture containing: 6 μl of 10× reaction buffer, 0.27 nkat trehalase (treF), 33 nkat glucose oxidase, 0.83 nkat horseradish peroxidase and 0.25 μl of 20 mM 10-acetyl-3,7-dihydroxyphenoxazine (Amplex Red^®^; dissolved in DMSO). After mixing, and centrifugation at 1000×*g* for 1 min, the resulting trehalase reaction was monitored using a Synergy^®^ HT microplate reader (BioTek, Winooski, VT, USA) in fluorescence mode (excitation 530 nm, emission 590) at 25°C for 30–40 min. The rate of each reaction was calculated using Gen5^®^ software (BioTek). The blank rate was subtracted from all measurements. An internal calibration curve was constructed from the rates of the reactions that had been supplemented with trehalose standards, and used to calculate the trehalose content of the non-supplemented sample.

### Sucrose and Tre6P determination

Sucrose was measured enzymatically in ethanolic extracts according to [93]. Tre6P was measured in chloroform-methanol extracts by high performance anion exchange liquid chromatography coupled to tandem mass spectrometry (LC-MS/MS) as described in Lunn et al. 2006 [32].

## Abbreviations

LC-MS/MS: Anion-exchange liquid chromatography-tandem mass spectrometry; TPP: Trehalose-phosphate phosphatase; TPS: Trehalose-phosphate synthase; Tre6P: Trehalose-6-phosphate.

## Competing interests

The authors declare that they have no competing interests.

## Authors’ contributions

MS and JL conceived the study. PC, RF and YG developed the trehalose assay and carried out the measurements. NSN and DJ planned the maize *ramosa3* mutant analysis and dissected the inflorescence primordia. OEB carried out the *A. thaliana* low temperature experiment. PC and JL drafted the manuscript and all authors approved the final manuscript.

## Supplementary Material

Additional file 1: Figure S1 Purification of the *Escherichia coli* cytoplasmic trehalase (treF). TreF was over-expressed in *E. coli* as a His_6_-tagged fusion protein and purified by immobilised metal affinity chromatography (Co^2+^, Talon™), size exclusion chromatography (Superdex S200) and anion exchange chromatography (Mono Q). Proteins in 0.5-2 μl aliquots of fractions from each stage of the purification were analysed by SDS polyacrylamide gel electrophoresis (10% gel) and stained with Coomassie Blue R250. M = molecular weight markers. Samples were: (1) *E. coli* cell lysate; (2) soluble cell extract; (3) Talon™ column pass through; (4) imidazole eluate from Talon™ column; (5–7) peak fractions from Superdex S200 column; (8–15) peak fractions from MonoQ column. Click here for file

Additional file 2: Figure S2 Specificity of the *E. coli* cytoplasmic trehalase (treF). Hydrolysis of trehalose, maltotriose and cellobiose by the *E. coli* cytoplasmic trehalase (treF) was determined by measuring the release of glucose in an end-point assay using glucose oxidase, peroxidase and Amplex Red^®^. The increase in fluorescence is expressed in arbitrary fluorescence units. Data are mean ± SD (*n* = 3). Click here for file
