# Supplementary material for: A fluorometric assay for trehalose in the picomole range
Source: Plant Methods. 2013 Jun 20;9:21. doi: 10.1186/1746-4811-9-21 (PMC3698175; doi:10.1186/1746-4811-9-21)
Supplement: Additional file 1: Figure S1 — Purification of the Escherichia coli cytoplasmic trehalase (treF). TreF was over-expressed in E. coli as a His6-tagged fusion protein and purified by immobilised metal affinity chromatography (Co2+, Talon™), size exclusion chromatography (Superdex S200) and anion exchange chromatography (Mono Q). Proteins in 0.5-2 μl aliquots of fractions from each stage of the purification were analysed by SDS polyacrylamide gel electrophoresis (10% gel) and stained with Coomassie Blue R250. M = molecular weight markers. Samples were: (1) E. coli cell lysate; (2) soluble cell extract; (3) Talon™ column pass through; (4) imidazole eluate from Talon™ column; (5–7) peak fractions from Superdex S200 column; (8–15) peak fractions from MonoQ column. [file 1746-4811-9-21-S1.pdf]

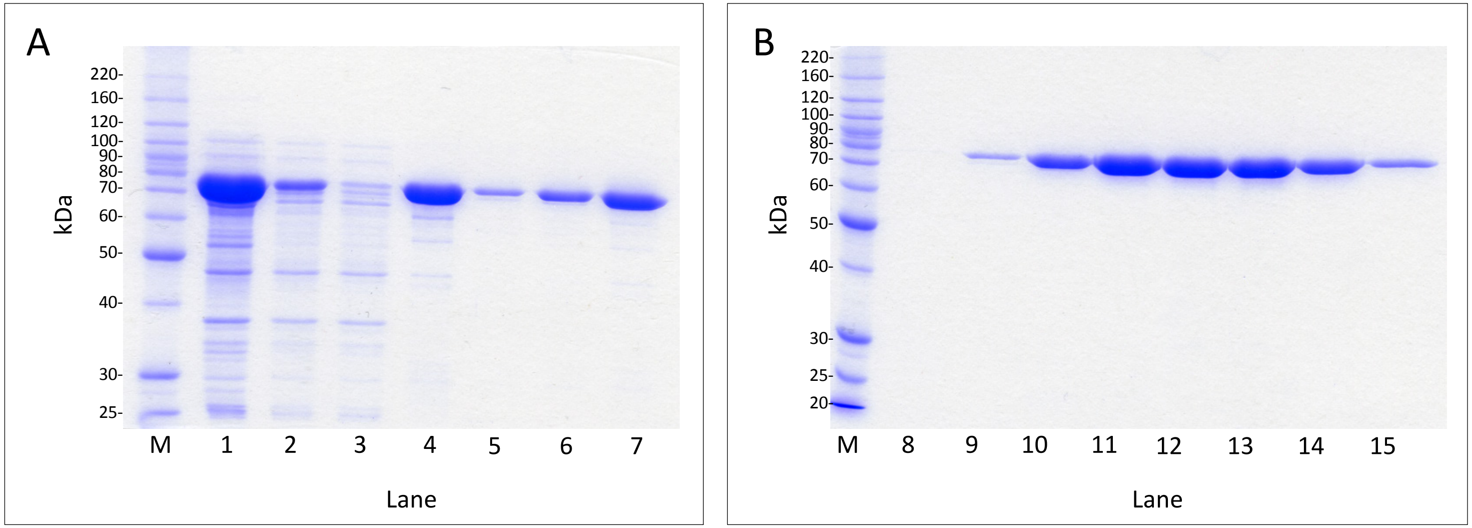

Supplemental Figure S1 Purification of the *Escherichia coli* cytoplasmic trehalase (treF). TreF was over-expressed in *E. coli* as a His_6-tagged fusion protein and purified by immobilised metal affinity chromatography (Co^{2+}, TalonTM), size exclusion chromatography (Superdex S200) and anion exchange chromatography (Mono Q). Proteins in 0.5-2 µl aliquots of fractions from each stage of the purification were analysed by SDS polyacrylamide gel electrophoresis (10% gel) and stained with Coomassie Blue R250. M = molecular weight markers. Samples were: (1) *E. coli* cell lysate; (2) soluble cell extract; (3) Talon^{TM} column pass through; (4) imidazole eluate from Talon^{TM} column; (5-7) peak fractions from Superdex S200 column; (8-15) peak fractions from MonoQ column.
