# Supplementary material for: A fluorometric assay for trehalose in the picomole range
Source: Plant Methods. 2013 Jun 20;9:21. doi: 10.1186/1746-4811-9-21 (PMC3698175; doi:10.1186/1746-4811-9-21)
Supplement: Additional file 2: Figure S2 — Specificity of the E. coli cytoplasmic trehalase (treF). Hydrolysis of trehalose, maltotriose and cellobiose by the E. coli cytoplasmic trehalase (treF) was determined by measuring the release of glucose in an end-point assay using glucose oxidase, peroxidase and Amplex Red®. The increase in fluorescence is expressed in arbitrary fluorescence units. Data are mean ± SD (n = 3). [file 1746-4811-9-21-S2.pdf]

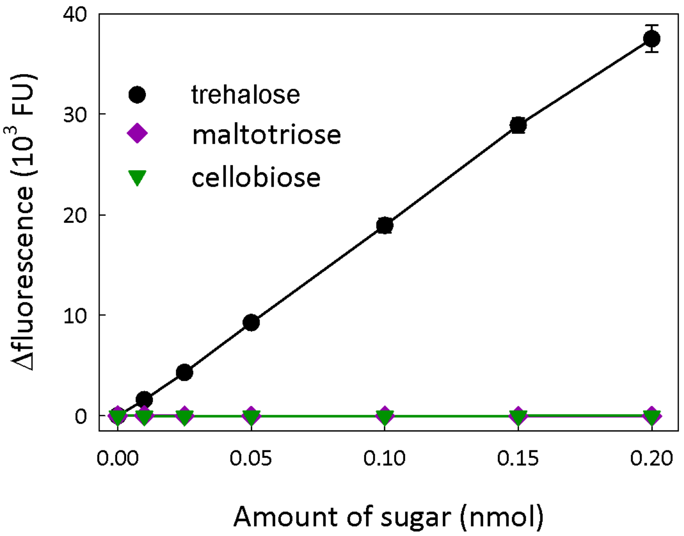

Supplemental Figure S2 Specificity of the *E. coli* cytoplasmic trehalase (treF). Hydrolysis of trehalose, maltotriose and cellobiose by the *E. coli* cytoplasmic trehalase (treF) was determined by measuring the release of glucose in an end-point assay using glucose oxidase, peroxidase and Amplex Red®. The increase in fluorescence is expressed in arbitrary fluorescence units. Data are mean ± SD (*n* = 3).
